# Process Optimization and Upscaling of Spray-Dried Drug-Amino acid Co-Amorphous Formulations

**DOI:** 10.3390/pharmaceutics11010024

**Published:** 2019-01-09

**Authors:** Georgia Kasten, Íris Duarte, Maria Paisana, Korbinian Löbmann, Thomas Rades, Holger Grohganz

**Affiliations:** 1 Department of Pharmacy, University of Copenhagen, Universitetsparken 2, 2100 Copenhagen, Denmark; georgia.kasten@sund.ku.dk (G.K.); korbinian.loebmann@sund.ku.dk (K.L.); thomas.rades@sund.ku.dk (T.R.); 2 Department of R&D Drug Product Development, Hovione FarmaCiencia SA, Estrada do Paço do Lumiar, Building S, 1649-038 Lisbon, Portugal; iduarte@hovione.com; 3 Department of R&D Analytical Chemistry Development, Hovione FarmaCiencia SA, Estrada do Paço do Lumiar, Building S, 1649-038 Lisbon, Portugal; mpaisana@hovione.com

**Keywords:** co-amorphization, spray drying, design of experiments, pharmaceutical development, particle engineering

## Abstract

The feasibility of upscaling the formulation of co-amorphous indomethacin-lysine from lab-scale to pilot-scale spray drying was investigated. A 2^2^ full factorial design of experiments (DoE) was employed at lab scale. The atomization gas flow rate (F_atom_, from 0.5 to 1.4 kg/h) and outlet temperature (T_out_, from 55 to 75 °C) were chosen as the critical process parameters. The obtained amorphization, glass transition temperature, bulk density, yield, and particle size distribution were chosen as the critical quality attributes. In general, the model showed low F_atom_ and high T_out_ to be beneficial for the desired product characteristics (a co-amorphous formulation with a low bulk density, high yield, and small particle size). In addition, only a low F_atom_ and high T_out_ led to the desired complete co-amorphization, while a minor residual crystallinity was observed with the other combinations of F_atom_ and T_out_. Finally, upscaling to a pilot scale spray dryer was carried out based on the DoE results; however, the drying gas flow rate and the feed flow rate were adjusted to account for the different drying chamber geometries. An increased likelihood to achieve complete amorphization, because of the extended drying chamber, and hence an increased residence time of the droplets in the drying gas, was found in the pilot scale, confirming the feasibility of upscaling spray drying as a production technique for co-amorphous systems.

## 1. Introduction

It has been estimated that between 80% and 90% of new chemical entities in the research and development pipelines are poorly water-soluble [1], being classified as class II or IV in the Biopharmaceutics Classification System (BCS) [2]. This implies solubility as a limiting step for oral absorption, and thus a bioavailability and pharmacological effect [3]. The administration of drugs in an amorphous form has been shown to be a possible approach to address the challenges related to this poor solubility. As an amorphous form exhibits a higher internal energy and reactivity than its crystalline counterpart, the drug becomes potentially more soluble, and thus more easily absorbed [4]. However, the amorphous form of drugs can be physically unstable. As one option to address this instability, the use of co-amorphous formulations has gained interest in the past decade [3,5,6]. These systems are glass solutions representing a single phase (homogeneous) amorphous system of two or more low-molecular weight, initially crystalline components, namely: a combination of two pharmacologically relevant drugs [7,8,9] or a combination of a drug with a low molecular weight excipient [10,11,12,13,14]. The excipient is in this case called the co-former, and promotes the stabilization of the drug in the desired amorphous form. Several studies report on the potential of amino acids (AA) as co-formers for increased physical stability [15,16,17].

In general, co-amorphous formulations can be prepared by techniques such as ball milling (BM), spray drying, freeze drying, quench cooling, hot melt extrusion, or solvent evaporation [11,18]. BM can be useful to assess the co-formability between the drug–AA pair, and to obtain information on the *Tg* of the resulting co-amorphous formulation. Whilst BM is very useful for screening purposes on a laboratory scale, only a few publications delve into the preparation of co-amorphous formulations using larger scalable techniques, so that their potential can be explored industrially. The manufacturing feasibility of the arginine–indomethacin pair by hot melt extrusion (HME) was studied by Lenz et al. [19]. However, the process appeared to be complex because of the high melting points of the AA, which caused an uneven amorphization of the product, and phase separation. The difficulty in melting AAs because of their degradation limits the use of the HME technique [19]. Spray drying (SD) is a scalable technique based on solvent evaporation and is characterized by rapid heat and mass transfer between the sprayed droplets and the drying gas [20]. However, the transfer from a dry technique, such as BM, towards a solvent technique, such as SD, requires the consideration of the influence of the moisture present during production on the obtained product. In this respect, previous work by our group reported an interesting drug–AA formulation of indomethacin and lysine (IND–LYS), in which different physical forms of the salt were produced, depending on the presence of moisture during BM [21]. The XRPD analysis indicated that a co-amorphous product was obtained upon dry BM, and a crystalline product was obtained by BM with 1 g of a IND–LYS powder mixture with 50 µL of water. Salt formation was confirmed in both cases by FTIR. The co-amorphous salt showed an excellent physical stability and significant increase in the intrinsic dissolution rate compared to amorphous IND, crystalline IND, and the crystalline form of the salt. The powder dissolution experiments also showed the potential of the co-amorphous IND–LYS salt to supersaturate. Hence, the moisture present during the processing can influence the physical form and in turn the performance of the product.

The rapid heat and mass transfer during SD is assumed to be an advantage when co-amorphization is desired, as the drug molecules will not have time to recrystallize during the solvent drying [22]. Co-amorphous salt formulations of the acidic drug indomethacin with basic amino acids (arginine, histidine, and lysine) were successfully prepared by lab scale SD, even though the indomethacin–histidine salt could not be prepared by BM [23]. In another lab scale SD study, variations in the solvent composition were shown to affect the success in amorphization between the basic drug carvedilol and the acidic amino acids (aspartic acid and glutamic acid) [24]. Therefore, SD seems to be an interesting technique to produce drug–AA co-amorphous formulations, however, the upscaling of these formulations has not yet been investigated.

In the current work, SD was evaluated as a method for the preparation of IND–LYS co-amorphous formulations at a laboratory and pilot scale. The feasibility of the upscaling was based on a screening design of experiments (DoE) of the critical process parameters atomization gas flow rate (F_atom_) and outlet temperature (T_out_) in a laboratory scale spray dryer. F_atom_ and T_out_ were varied to observe the effect of the droplet size and the drying kinetics/efficiency on co-amorphization. Finally, the most promising formulation was scaled up to a pilot scale spray dryer. To account for the different dryer geometry, the feedstock solution flow rate and drying gas flow rate were doubled, while the remaining laboratory scale conditions were maintained. The products were compared not only with regard to amorphization, but also to the glass transition temperature (*Tg*), yield, bulk density, and particle size distribution.

## 2. Materials and Methods

### 2.1. Materials

Indomethacin (IND) was purchased from Fagron Nordic AS (Copenhagen, Denmark), and L-lysine (LYS) was purchased from Sigma Aldrich (St. Louis, MO, USA). All of the substances were of reagent grade and were used as received. Acetone was purchased from Drogas Vigo, S.L. (Porriño, Spain), and ultrapure water was obtained by a Elix Advantage Purification System (Merck Millipore KGaA, Darmstadt, Germany).

### 2.2. Methods

#### 2.2.1. Preparation of Co-Amorphous Formulations at Laboratory Scale SD

##### 2.2.1.1. Feedstock Solution Preparation

The feedstock solution for the SD process consisted of a mixture of an organic solvent and ultrapure water, so that the poorly water-soluble drug, IND, and the water-soluble AA, LYS, could be successfully dissolved. This solution was optimized from Jensen et al. [23]. Given that the organic solvents exhibit a higher vapor pressure, the maximum organic solvent to water ratio was employed, thereby permitting a faster solvent evaporation. Then, 1010 g of the solvent mixture (acetone:water 53:47 (*w*/*w*)) was added to 10 g of IND and LYS weighed in an equimolar ratio, so that the final concentration of solids was 0.98% (*w*/*w*%). Subsequently, this mixture was ultrasonicated into a clear solution suitable for the SD process.

##### 2.2.1.2. Spray Drying at Laboratory Scale

A Mini Spray Dryer B-290 (Büchi Labortechnik AG, Flawil, Switzerland) equipped with an inert loop B-295 (Büchi Labortechnik AG) was used. The SD was conducted with a two-fluid nozzle (Ø0.7/1.5 mm) with water cooling, under a nitrogen drying gas flow of 25 kg/h (100% aspiration), and at a feedstock solution flow rate of 0.5 kg/h. The atomization gas flow rate and the outlet temperature were varied (0.5 to 1.4 kg/h, and 55 to 75 °C, respectively) following a full 2^2^ factorial design with a center point, as described in Section 2.2.1.3. The SD process lasted around 2 h, and the formulation powder was collected in amber glass vials. Furthermore, the product was weighed and the total yield (%) was calculated as the mass percentage of the SD product in relation to the total solids in the spray dryer feedstock solution. Afterwards, a secondary tray drying step was applied as a customary industrial procedure to ensure that the solvent residues were at a sufficiently low level. Tray drying was applied for 48 h in a 30 °C oven under vacuum, after an initial nitrogen sweep.

##### 2.2.1.3. Experimental Design

A 2^2^ full factorial design with one center point was employed to investigate the influence of F_atom_ and T_out_ on the final material’s attributes (*viz*. amorphousness, *Tg*, yield, bulk density, and particle size distribution). The DoE performed is described in Table 1. It is important to notice that the T_out_ chosen for this design was below the *Tg* of the ball milled co-amorphous reference formulation (*Tg* = 100.5 °C) described in the literature [21].

The analytical results obtained were used as responses and were analyzed with Modde Pro software (MKS Instruments, Andover, MA, USA). The degree of amorphization was calculated with X’Pert Highscore Plus software (PANalytical, Almelo, the Netherlands) from the area under the curve of the diffractograms, after the background subtraction. The co-amorphization was attributed to a sample when an amorphous halo and a single *Tg* were found [25]. The model was fitted using a multiple linear regression (MLR) and was optimized by the deletion of non-significant terms. Thus, the exclusion was based on the coefficients plot for each variable, given the respective significance, and also on the quality of the model after deletion. Afterwards, the results were summarized in contour plots so as to identify the best drying conditions for co-amorphization and upscaling.

#### 2.2.2. Analytical Techniques

##### X-ray Powder Diffraction (XRPD)

The XRPD measurements were performed using an Empyrean X-ray diffractometer (PANalytical, Almelo, The Netherlands) with CuKα radiation (1.54 Å), and an acceleration voltage and current of 45 kV and 40 mA, respectively. The samples were scanned in reflectance mode between 3° and 35° 2θ, with a scan speed of 0.033667° 2θ/s, a step size of 0.0131°, and 100 s per step. The data were collected and analyzed using the software X’Pert Data Collector (PANalytical, Almelo, The Netherlands).

##### Differential Scanning Calorimetry (DSC)

DSC measurements were performed using a DSC Discovery 250 (TA Instruments, New Castle, DW, USA). Powder samples of approximately 2–3 mg were weighed in Tzero aluminum pans and were closed with Tzero standard lids. The analyses were conducted under a nitrogen flow of 50 mL/min, in the modulated temperature mode, with a heating rate of 2 °C/min, amplitude of 0.21 °C, and period of 40 s, until 180 °C, after an isothermal step of 1 min at −10 °C. The experimental glass transition temperatures (*Tg*, midpoint) were determined from the reversing heat flow signal of three replicates using Trios v4.3.1.39215 software (TA Instruments, New Castle, DW, USA).

##### Bulk Density

The volume was estimated after weighing the powder samples into 25 mL graduated cylinders. The bulk density was then calculated in g/cm^3^ [26].

##### Particle Size Distribution

The particle size distribution and span of the SD formulations were analyzed with a Helos/BR laser diffraction sensor, coupled with an Aspiros sample feeder module and Rodos/M dry dispersion unit (Sympatec GmbH, Clausthal-Zellerfeld, Germany). The particles were dispersed at 18 mm/s in a compressed air flow, under a pressure of 6.5 bar, and focused with an R2 lens (range of 0.25 to 87.5 µm). The volume-weighted size distributions were estimated using the Helos Sensor Control software (Sympatec GmbH, Clausthal-Zellerfeld, Germany) using the Fraunhofer theory, and the particle diameters of *d*_10_, *d*_50_, and *d*_90_ were calculated, expressing that 10%, 50%, and 90% of the particles were smaller than the given values. The span value was also estimated by the software (calculated via *d*_90_–*d*_10_/*d*_50_). All of the measurements were made in triplicate.

#### 2.2.3. Upscaling from Laboratory Scale to Pilot Scale SD

The laboratory scale formulation with the highest potential for co-amorphization was chosen for upscaling. In this experiment, the same feedstock solution as in Section 2.2.1.1 was used. As for the laboratory scale process, the pilot scale spray drying equipment was operated in closed-loop with a condenser and a two-fluid nozzle (Ø0.7/1.5 mm) with water cooling. Because of the approximately 30 times larger drying chamber of this unit, the nitrogen drying gas flow was 43 kg/h and the feedstock solution flow rate was 1.0 kg/h, for example, approximately double the laboratory scale SD. In addition, the relative saturation at the outlet of the drying chamber was calculated using a thermodynamic model, as described in detail by Gaspar et al. ([27], p. 275).

## 3. Results

### 3.1. Characterization of Formulations Produced in a Laboratory Scale Spray Dryer

**Figure 1:**
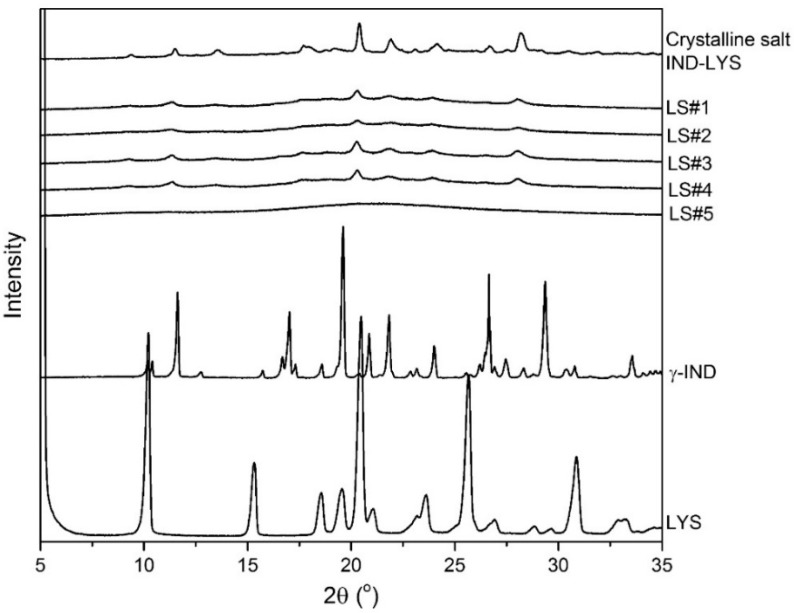
XRPD diffractograms of indomethacin and lysine (IND–LYS) formulations #1 to #5 prepared in laboratory scale spray dryer, the crystalline initial materials, and the crystalline salt of IND–LYS described previously [21].

The formulations produced in the laboratory scale spray dryer were characterized with regard to their amorphousness, *Tg*, yield, bulk density, and particle size distribution. The results are shown in Figure 1 and Table 2. The effect of the process parameters of F_atom_ and T_out_ will be discussed in Section 3.2. First, the solid state of the formulations was investigated by XRPD. The diffractograms of the powder formulations are shown in Figure 1, showing the crystalline initial materials and the final powder formulations.

The spray drying of IND–LYS resulted in either partly crystalline or in amorphous formulations, depending on the drying conditions employed. In Figure 1, it can be observed that the formulations #1 to #4 show some remaining crystallinity diffractions, while formulation #5 exhibited only an amorphous halo. Furthermore, a more detailed view of the diffractograms in comparison to the initial materials (crystalline IND and LYS) indicates that formulations LS#1 to #4 are in fact the crystalline salt of IND-LYS [21].

The thermal analysis of the formulations revealed *Tg*s between 96.2 (±0.2) °C and 102.7 (±4.4) °C, indicating that even though formulations #1 to #4 presented crystalline reflections, they contained also an amorphous fraction (Table 2). The experimental *Tg* found for spray dried IND-LYS formulations is in line with the *Tg* of the IND-LYS co-amorphous formulation described in the literature (100.5 °C) [21].

The resulting yield was between approx. 50% and 97% (Table 2). This variation may be related to the process parameters resulting in sticking of the powder to the cyclone walls [28]. The bulk density was determined for all the formulations, and the results showed that it varied between 0.25 and 0.37 g/cm^3^, indicating that the droplets’ drying kinetics during the spray drying process resulted in particles exhibiting higher or lower density. Regarding the particle size, the mean diameter (*d*_50_) varied between 2.71 and 4.23 µm, and the obtained particles presented a monomodal population, with rather wide distribution, given the span values (Table 2).

### 3.2. Model Statistics

**Figure 2:**
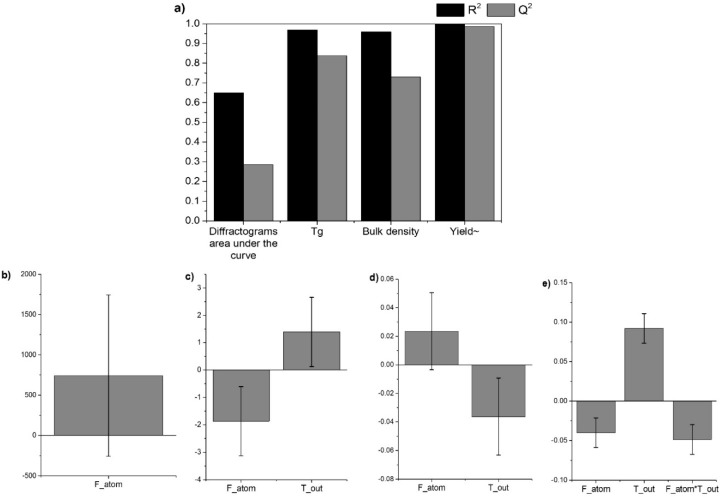
(**a**) R^2^ and Q^2^ values obtained after model optimization. Coefficient plots displaying the scaled and centered regression coefficients with confidence intervals (confidence level 0.95), for the adjusted model after transformation (if applicable) and variable selection. Responses: (**b**) area under the diffractograms curve, (**c**) *Tg*, (**d**) bulk density, and (**e**) yield.

The DoE systematically varies the input factors, while simultaneously evaluating their effect and the interactions on the responses, in contrast to the one factor at a time studies [29,30,31]. In this work, the DoE was used to investigate the effect of the F_atom_ and T_out_ on amorphization, *Tg*, yield, bulk density, and particle size distribution. The resulting R^2^ and Q^2^ values, which represent the goodness of fit and goodness of prediction of the model, respectively, can be observed for each response in Figure 2a. The coefficients plots are shown in Figure 2b–e.

Figure 2a shows that high values of R^2^ were obtained (0.65–1.00), indicating that the model successfully explains the data. The Q^2^ values, however, varied between 0.98 for the yield to 0.28 for the area under the curve of the diffractograms, meaning that the model has a limited prediction capacity for different responses. It should be mentioned that the particle size response had a R^2^ = 0.24. As all of the values were in a desired area (small particles, 2–4 µm, Table 2) for all of the formulations, no statistical influence of the process parameters was found in the investigated range. The variations in the closeness between R^2^ and Q^2^, as well as the actual values found, indicate that the underlying mathematical relations for the responses differ. Whilst the full factorial design assumes a linear influence of the input variables, this assumption might not be valid for all of the responses. From a mathematical point-of-view, a lack of fit can be addressed by the application of more evolved models such as the central composite designs, which would include non-linear model terms. From a practical perspective, however, one might also conclude that the process parameters did not significantly influence the responses, thus, indicating a robust production process.

The optimized coefficient plots for the responses are shown in Figure 2b–e. The coefficient plot for the response diffractograms area under the curve (Figure 2b) shows F_atom_ as being non-significant, despite its high coefficient. Hence, this factor was maintained to preserve the fit (R^2^ = 0.65). This plot also indicates that there might be a positive tendency of an increase in the crystallinity when increasing F_atom_. The diffractograms area under the curve, however, may not be the ideal method for the discrimination of crystalline and amorphous content, even though it is a useful method for comparison [32]. Individual areas of the Bragg peaks were also used in an attempt to improve the model, but with no success (data not shown).

Figure 2c shows the *Tg* response, which was significantly influenced by both of the factors present in the model. A slight increase in *Tg* can be observed when decreasing F_atom_ and increasing T_out_. Even though the difference between the lowest and highest *Tg*s found experimentally was not substantial (96–102 °C, Table 2), it can be inferred that an increase in *Tg* was observed when the process was held at a high T_out_. This can be explained by a higher T_out_ providing faster drying kinetics [27], thereby preventing the remaining water from acting as a plasticizer and decreasing the *Tg* value [33].

Figure 2d shows the coefficient plot for the response bulk density. Both of the process parameters affect the bulk density response. F_atom_, however, was depicted as non-significant, but was maintained in the model to preserve the model quality (R^2^ = 0.96). Higher density values were obtained when increasing F_atom_ and decreasing T_out_. This indicates that a more dense powder was obtained with small droplets that were slowly dried. This is expected, as such conditions can lead to shriveled particles [34].

Figure 2e shows the coefficient plot for the response yield after performing the logarithmic transformation of the raw data, because of skewedness. Upon transformation, the yield exhibited a R^2^ = 1.00, and the model indicated F_atom_, T_out_, and the interaction between those factors as significant. An increase in the yield tends to occur when lowering the F_atom_ and increasing T_out_. This is probably due to the fact that bigger particles were produced with a low F_atom_, which were then easily collected by the cyclone. Also, better dried particles were produced with a high T_out_, leading to less stickiness of the powder inside the equipment, and therefore a higher yield [34,35].

### 3.3. Overall Model

**Figure 3:**
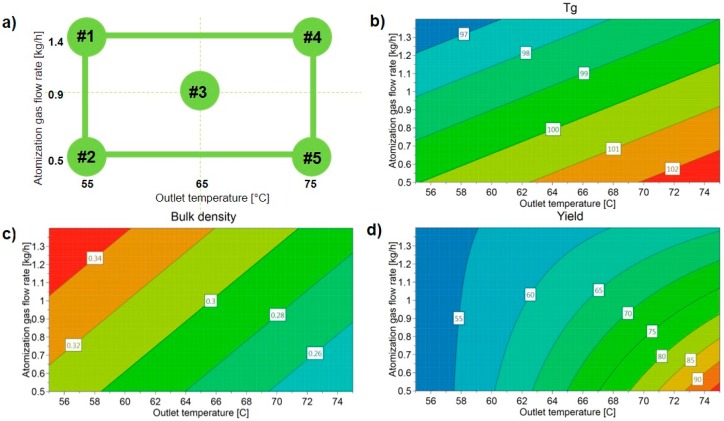
(**a**) Experimental design space, with LS formulation numbers; contour plots made with Modde Pro software for (**b**) *Tg* (°C); (**c**) bulk density (g/cm^3^); (**d**) yield (%).

The optimized model for all of the factors and responses shows the influence of the process parameters on the final product, allowing for the selection of the most feasible conditions for obtaining the (co-amorphous) product with a desired quality (high *Tg*, low bulk density, and high yield). Figure 3a shows the schematic of the formulations’ design space, while the influence of the varied factors on each single response is presented in contour plots in Figure 3b–d.

Figure 3b–d indicates that the most suitable conditions for such a product are combined around a low F_atom_ and high T_out_. This is shown by Figure 3b, as the highest *Tg* can be found in the bottom right corner of the design space, as well as the lowest bulk density (Figure 3c) and highest yield (Figure 3d). The combination of a low F_atom_ and high T_out_ (relative to formulation LS#5) is therefore shown by the model as paramount for obtaining the product with the desired quality attributes. Moreover, Formulation LS#5 exhibited an amorphous halo on the XRPD analysis (Figure 1), and therefore its processing conditions were chosen for the upscale.

### 3.4. Upscaling of Selected Formulations to Pilot Scale Spray Dryer

**Figure 4:**
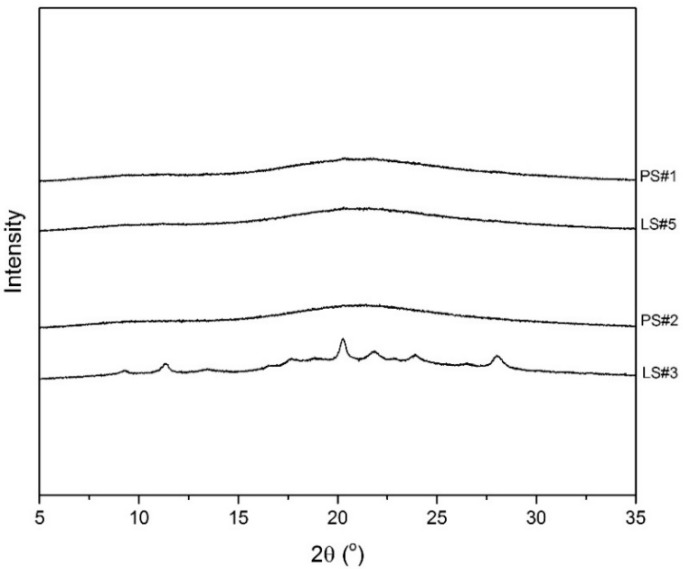
XRPD measurements comparing the outcome of the laboratory scale spray dried formulations to the respective formulations produced in the pilot scale spray dryer during upscaling.

The upscaling of the co-amorphous IND–LYS was implemented after the DoE at a laboratory scale. As a low F_atom_ and high T_out_ were found to be the most suitable settings to reach a co-amorphous IND–LYS formulation by SD, Formulation LS#5 was chosen for the upscaling (PS#1). In addition, the center point (formulation LS#3) was included in the upscaling (PS#2). This formulation, produced with an intermediate F_atom_ and T_out_, could potentially benefit from the superior drying capacity, given the increased drying gas flow rate and longer residence time in an extended drying chamber (both linked to better water evaporation capacity, and lower relative saturation of the drying gas [27]). Figure 4 shows the XRPD diffractograms of the formulations obtained in both scales (LS and PS), while Table 3 shows the *Tg*, bulk density, yield, and particle size distribution for the formulations produced in the pilot scale spray dryer.

In contrast to the laboratory scale samples, both of the formulations became amorphous in the pilot scale spray dryer, as can be seen by the amorphous halos observed in Figure 4. Also, a single *Tg* was measured by DSC in each formulation (Table 3), confirming the co-amorphous nature of the products. Furthermore, the *Tg*s were in line with the formulations produced by the laboratory scale spray drying process. In addition, the obtained yield and bulk density were also comparable to the laboratory scale process (Table 2 and Table 3). The mean particle size (*d*_50_) obtained for both of the formulations produced by the pilot scale spray dryer, however, appeared to be slightly reduced, even though the same nozzle and F_atom_ was used for the atomization and formation of the droplets. This suggests that the larger dimensions of the drying chamber also decreased the collisions in-between the droplets and with the walls of the equipment. The reduction of the likelihood of collisions could avoid a process-induced increase in *d*_50_ [27,36].

Furthermore, during the upscale, the drying gas flow rate was doubled, because of the larger dimensions of the pilot scale apparatus. Although the feedstock flow rate also was doubled, the relative saturation of the water and organic solvent in the gas reentering the system were calculated to be lower compared with the laboratory process. This means that the drying of the droplets into solid particles occurred faster, and thereby increased the likelihood for the formation of an amorphous product [27,36]. In this context, the upscaling of co-amorphous IND–LYS formulations was successful, and seemed to be more straightforward once the drying conditions were understood. The current study could form the basis for an in-depth investigation to elucidate the influence of the process and formulation parameters at a pilot scale level, if such was desired. It might then be considered to apply a response surface model (such as a central composite design) that would also cover non-linear terms. Furthermore, as the laboratory scale DoE showed the optimum in a corner of the design, it might be worth considering a relocation of the design space to investigate even lower F_atom_ and higher T_out_ settings.

With regard to further upscaling to a manufacturing scale, further investigation would be required, as the critical process and formulations parameters, such as the dimensions of the drying chamber, nozzle type, feed flow rate, and relative saturation of the gas, are likely to be altered. Nevertheless, the current study showed that the spray-drying of the co-amorphous formulations can be scaled up from a laboratory to pilot scale. The observation that even previously suboptimal conditions were applicable in a pilot scale indicates that further upscaling should be feasible.

## 4. Conclusions

The feasibility of upscaling the formulation of the co-amorphous IND–LYS from laboratory scale to pilot scale spray drying was investigated. A DoE was employed to identify the influence of two of the critical process parameters (atomization gas flow rate and outlet temperature, F_atom_ and T_out_, respectively) on the critical quality attributes (amorphousness, *Tg*, bulk density, yield, and particle size distribution). The conditions of a low F_atom_ and high T_out_ were selected for the upscaling, given that these conditions lead to a co-amorphous product with the highest *Tg*, lowest bulk density, and highest yield. Furthermore, the co-amorphization seemed to be easier when processing the co-amorphous drug–amino acid formulations in a higher scale, presumably because of the superior drying capacity of the larger apparatus.

## Figures and Tables

**Table 1:** Design of experiments (DoE) levels and corresponding actual experimental conditions of F_atom_ and T_out_ for laboratory scale (LS) spray drying of indomethacin and lysine (IND–LYS).

Formulation	Levels (F_atom_, T_out_)	
**LS#1**	(+, −)	1.4 kg/h, 55 °C
**LS#2**	(−, −)	0.5 kg/h, 55 °C
**LS#3**	(0, 0)	0.9 kg/h, 65 °C
**LS#4**	(+, +)	1.4 kg/h, 75 °C
**LS#5**	(−, +)	0.5 kg/h, 75 °C

**Table 2:** Experimental values found for the glass transition temperature (*Tg*), yield, average bulk density, and particle size distribution for all five of the laboratory scale (LS) produced formulations. SD—standard deviation.

Formulation	T_g_ (°C) (±SD)	Yield (%)	Bulk Density (g/cm^3^) (±SD)	Particle Size Distribution	
				*d*_50_ (µm) (±SD)	Span (±SD)
**LS#1**	96.16 (±0.18)	52.7	0.37 (±0.01)	2.88 (±0.69)	6.81 (±0.18)
**LS#2**	100.34 (±3.20)	50.6	0.31 (±0.01)	2.12 (±0.01)	4.47 (±0.22)
**LS#3**	99.04 (±0.67)	65.0	0.29 (±0.01)	4.23 (±0.36)	3.71 (±0.22)
**LS#4**	99.35 (±1.25)	64.4	0.28 (±0.01)	2.71 (±0.03)	6.03 (±0.10)
**LS#5**	102.72 (±4.44)	96.8	0.25 (±0.01)	3.19 (±0.12)	2.33 (±0.02)

**Table 3:** Experimental values found for *Tg*, yield, bulk density, and particle size distribution, for pilot scale (PS) produced formulations.

Formulation	Average T_g_ (°C) (±SD)	Yield (%)	Bulk Density (g/cm^3^) (±SD)	Particle Size Distribution	
				*d*_50_ (µm) (±SD)	Span (±SD)
**PS#1**	103.16 (±4.99)	83.35	0.24 (±0.01)	2.56 (±0.07)	3.01 (±0.21)
**PS#2**	99.66 (±0.14)	73.15	0.25 (±0.01)	1.97 (±0.02)	2.38 (±0.35)
